# Supplementary material for: TMEM33 regulates intracellular calcium homeostasis in renal tubular epithelial cells
Source: Nat Commun. 2019 May 2;10:2024. doi: 10.1038/s41467-019-10045-y (PMC6497644; doi:10.1038/s41467-019-10045-y)
Supplement: Supplementary file 1 — Supplementary Information [file 41467_2019_10045_MOESM1_ESM.pdf]

TMEM33 regulates intracellular calcium homeostasis in renal tubular epithelial cells

Arhatte et al.,

Supplementary Figure 1

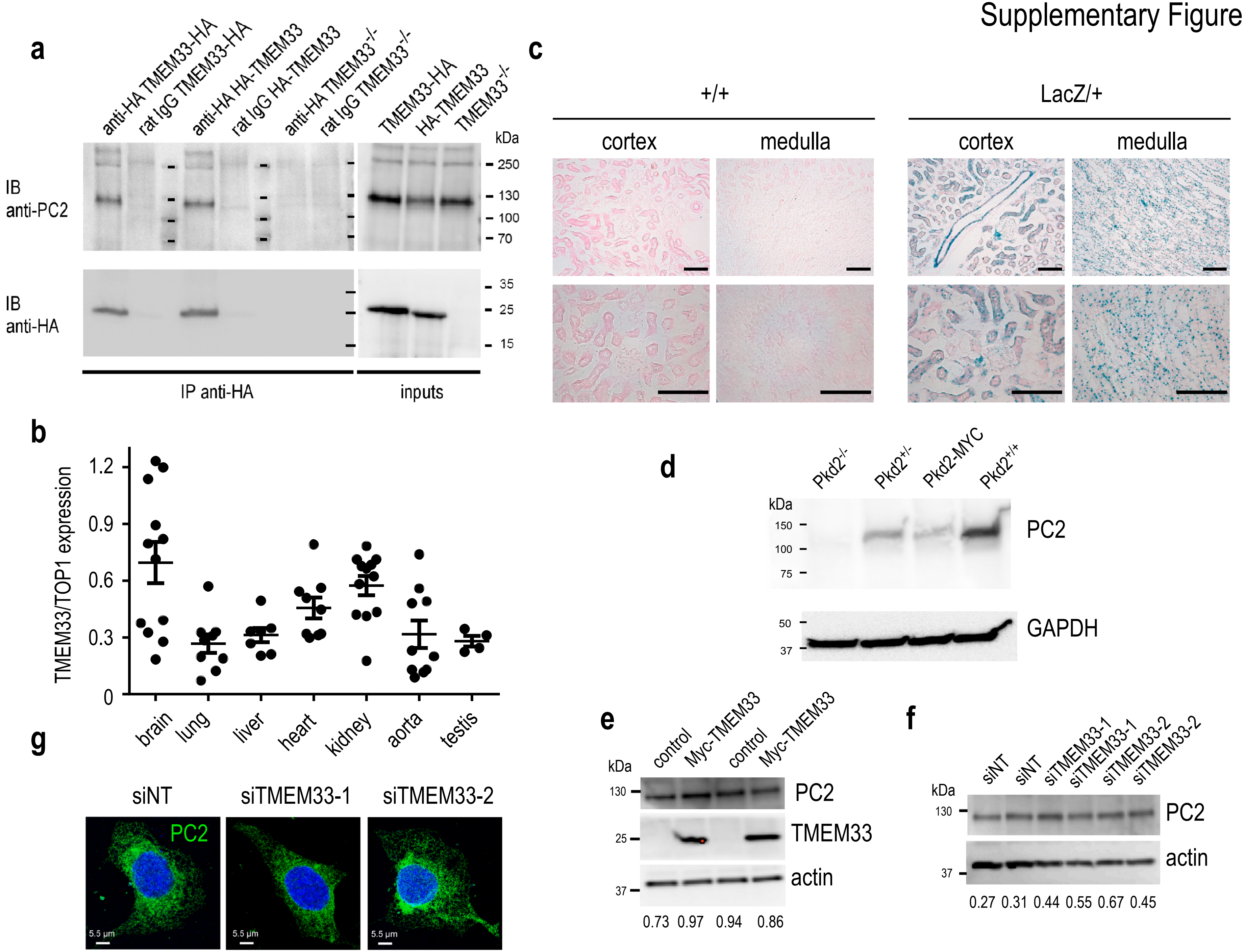

Supplementary Figure 1: TMEM33 expression, interaction with PC2 and knock-down. a) Co-immunoprecipitation of TMEM33 with PC2. TMEM33-HA or HA-TMEM33 are immunoprecipitated with an anti-HA antibody. In control experiments a rat IgG is used instead of the anti-HA antibody. Moreover, immunoprecipitation is absent with protein samples from TMEM33$^{-/-}$ cells. Inputs are shown on the right panel and antibodies used for immune blots are indicated on the left. **b)** Pattern of TMEM33 expression in mouse tissues, as detected by qPCR. Values are means ± SEM overlaid with dot plots. **c)** Expression of LacZ as a reporter of the TMEM33 promoter in the renal cortex and medulla of an heterozygote TMEM33$^{+/-}$ mouse (right panels). For comparison, lack of blue LacZ staining in WT kidneys is shown on the left panels. Scale bars indicate 200 μm. **d)** Validation of the E20 antibody directed against PC2 in PCT cell lines Pkd2$^{+/+}$, Pkd2$^{+/-}$, Pkd2$^{-/-}$ and Pkd2$^{-/-}$ complemented with *Pkd2*-MYC. **e)** Overexpression of Myc-TMEM33 or knock down of TMEM33 using siRNAs does not affect the expression of native PC2, as shown by Western blotting. Actin is used as a loading control. Normalized PC2/actin values are indicated at the bottom of the blots. **f)** Knock down of TMEM33 with siRNAs does not affect the expression of PC2 at the protein level. **g)** Knock down of TMEM33 with siRNAs does not affect the subcellular localization of PC2. In this experiment, PCT Pkd2$^{-/-}$ cells were transfected with PC2-MYC. PC2 is shown in green and the nucleus in blue (Hoechst staining). Source data are provided as a Source Data file.

Supplementary Figure 2

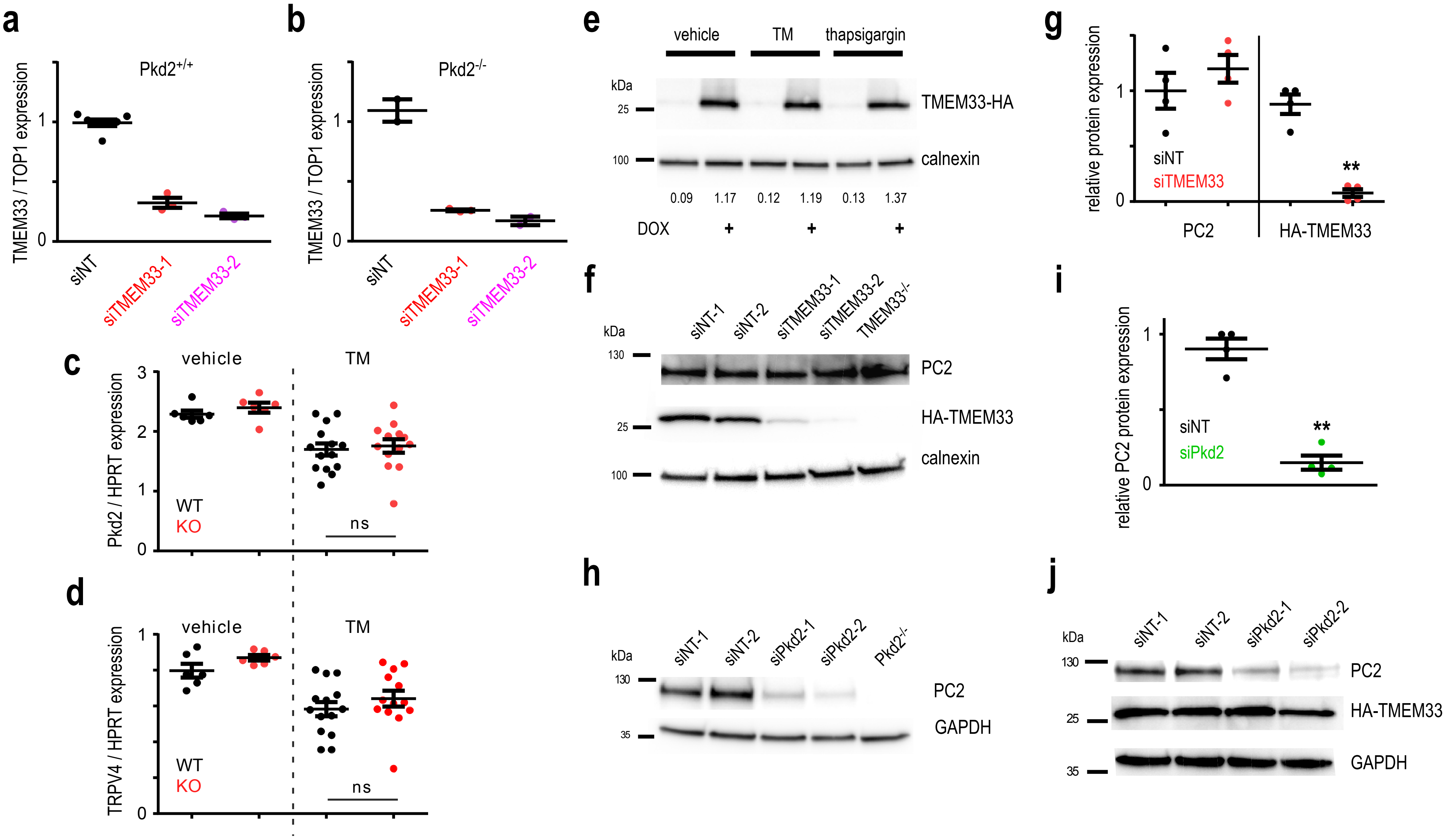

Supplementary Figure 2: **PC2 and TMEM33 knock-down validation**. **a)** Knock down of TMEM33 (red and magenta dots) compared to siNT (black dots), as detected by qPCR in WT PCT cells. **b**) Same in *Pkd2*$^{-/-}$ PCT cells. **c)** *Pkd2* expression versus HPRT in renal cortex from WT (black dots) and TMEM33 KO (red dots) mice injected with vehicle or TM. **d)** Same for TRPV4. **e)** ER stress induced by 1 μg/ml TM or 1 μM thapsigargin (16 hours) does not affect the level of expression of TMEM33-HA in a conditional PCT cells line, as compared to the vehicle condition. TMEM33-HA expression is induced by addition of DOX for 96 hours (as indicated) and calnexin is used as a loading control. Normalized TMEM33-HA/calnexin values are indicated at the bottom of the blot. **f)** Validation of siTMEM33 in PCT TMEM33$^{-/-}$ cells stably complemented or not with HA-TMEM33. **g)** Relative PC2 and TMEM33 expression in TMEM33$^{-/-}$ PCT cells stably complemented with HA-TMEM33 and transfected with control siNT (black dots) or siTMEM33 (red dots). **h**) Validation of siPkd2 in WT PCT cells. **i**) Relative PC2 expression in WT PCT cells transfected with control siNT (black dots) or siPkd2 (green dots). **j**) Knock-down of *Pkd2* does not impact TMEM33 protein expression in TMEM33$^{-/-}$ PCT cells stably complemented with HA-TMEM33 and transfected with control siNT (black dots) or siPkd2. Values are means ± SEM overlaid with dot plots. One star indicates $p < 0.05$, two stars $p < 0.01$ and three stars $p < 0.001$, with a one-way permutation test used to evaluate statistical significance. Source data are provided as a Source Data file.

Supplementary Figure 3

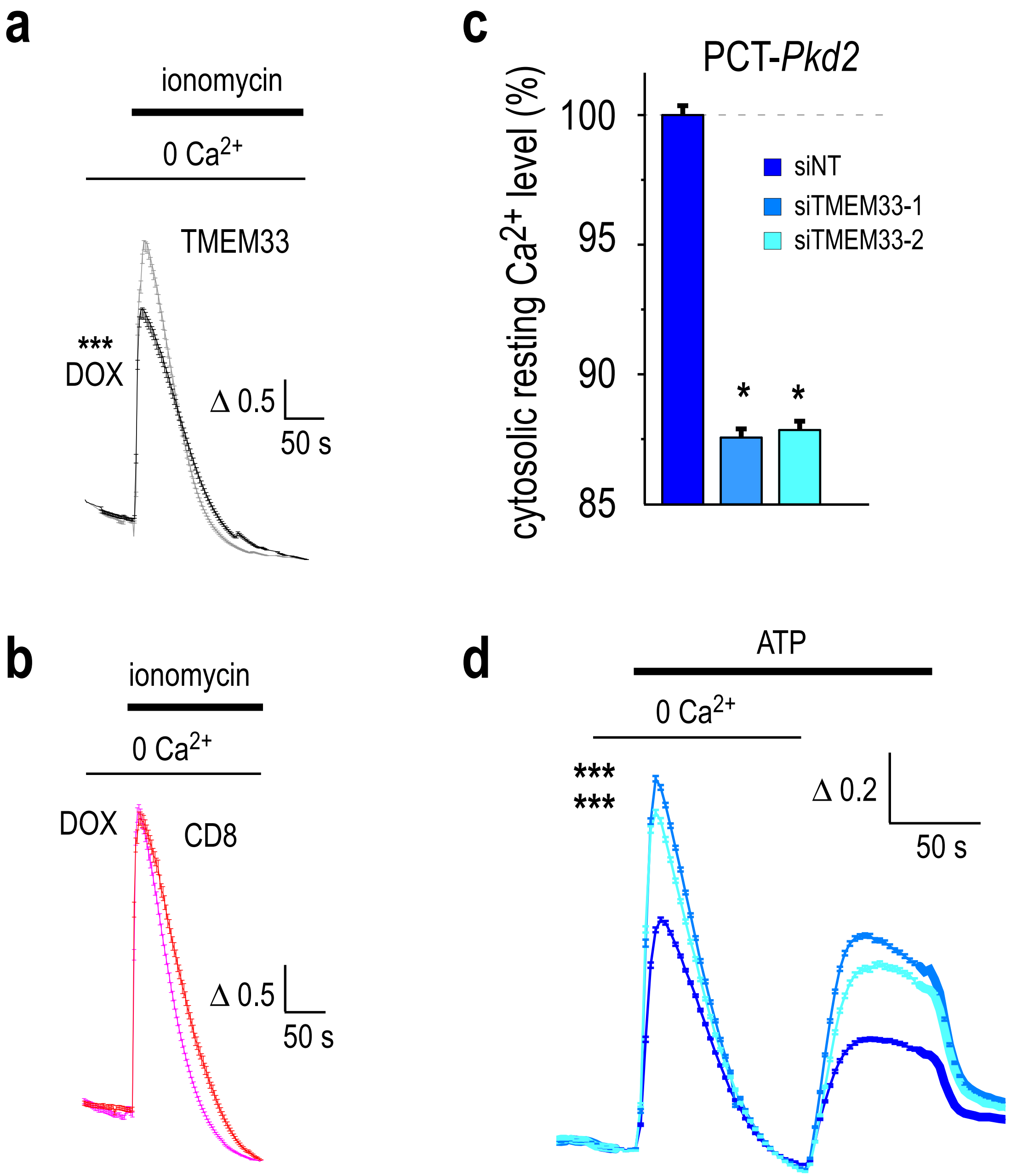

Supplementary Figure 3: TMEM33 influences ER calcium load. a) Calcium release induced by ionomycin in the absence of extracellular calcium in a conditional PCT cell line with enhanced TMEM33 expression in the presence of DOX (n=372, black trace) or not (n=423, gray trace). **b)** Same in a conditional PCT cell line expressing CD8ER (with DOX n=264, magenta trace; or not n =306, red trace). **c)** Increases in cytosolic calcium concentration are expressed as ratios of 340:380 nm fluorescence signals ($\Delta R/R_0$). Basal cytosolic calcium levels in a PCT cell line stably overexpressing PC2 and transfected with siNT (n= 2180; dark blue bar) or siTMEM33-1 (n= 2252; medium blue bar) or siTMEM33-2 (n=1821; light blue bar). Experimental points are available in the data source file. **d)** ATP calcium transients recorded in the absence of extracellular calcium in PCT cells stably overexpressing PC2 and transfected with siNT or siTMEM33-1 or siTMEM33-2 (Same cells as in panel a). Values are means ± SEM. One star indicates $p < 0.05$, two stars $p < 0.01$, three stars $p < 0.001$, with a Student's t-test used to evaluate statistical significance. Source data are provided as a Source Data file.

Supplementary Figure 4

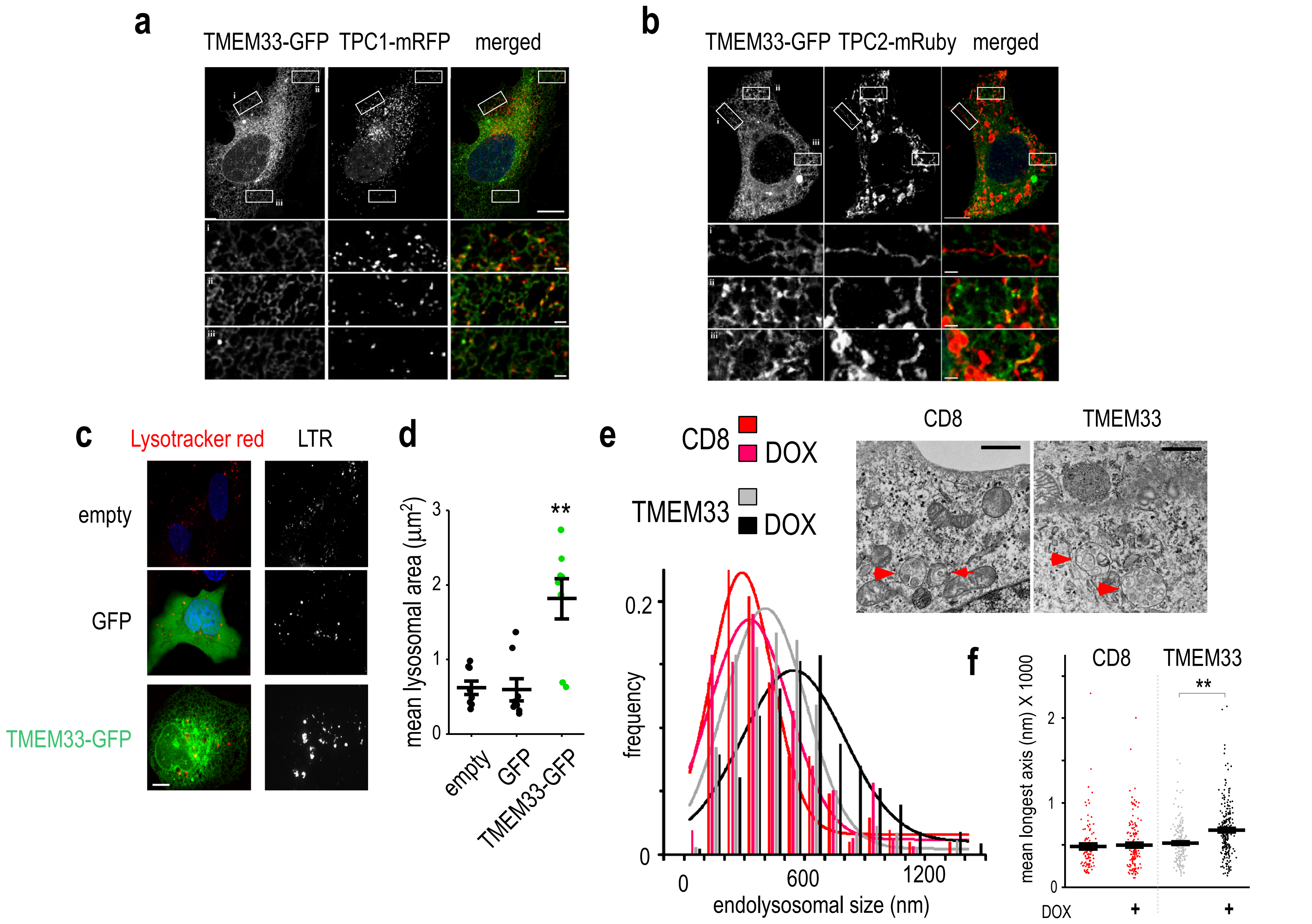

Supplementary Figure 4: TMEM33 causes lysosomal enlargement. a) Co-localisation of TMEM33-GFP (green) and TPC1-mRFP (red) in transiently transfected PCT cells. GFP and mRFP fluorescence is shown in black and white, while merged images are shown in color. Lower panels show magnifications of the boxed areas. Scale bar, 10 µm for full image, 1 µm for magnified regions of interest. **b)** Co-localization of TMEM33-GFP (green) and TPC2-mRuby2 (red) in transiently transfected PCT cells. Scale bar, 10 µm for full image, 1 µm for magnified regions of interest. **c**) Effect of TMEM33-GFP overexpression (in green), as compared to empty vector or GFP conditions in WT PCT cells on lysosomal size determined with Lysotracker (in red). Right panels show thresholded Lysotracker Red (LTR) in black and white. Scale bar is 10 µm. **d**) Dot plot shows quantification of the mean individual lysosomal area. Lysosome morphology was quantified from 8 images each (60 cells analyzed), collected across 3 independent transfections. Black dots: empty transfection and GFP; green dots: TMEM33-GFP. **e**) Electron microscopy pictures showing endolysosomes in DOX-induced conditional PCT cells expressing CD8ER (red: uninduced; magenta: induced with DOX) or TMEM33 (gray: uninduced; black induced with DOX). Lower left histogram shows the distribution of endolysosomal size. Scale bar is 2 µm. **f**) Dot plot showing quantification of the mean longest axis in TMEM33$^{-/-}$ PCT cell lines expressing CD8ER (red dots) without or with DOX or expressing TMEM33 without (gray dots) or with DOX (black dots). Values are means ± SEM overlaid with dot plots for d and f. One star indicates $p < 0.05$, two stars $p < 0.01$ and three stars $p < 0.001$, with two-tailed Student's t-test used to evaluate statistical significance. Source data are provided as a Source Data file.

Supplementary Figure 5

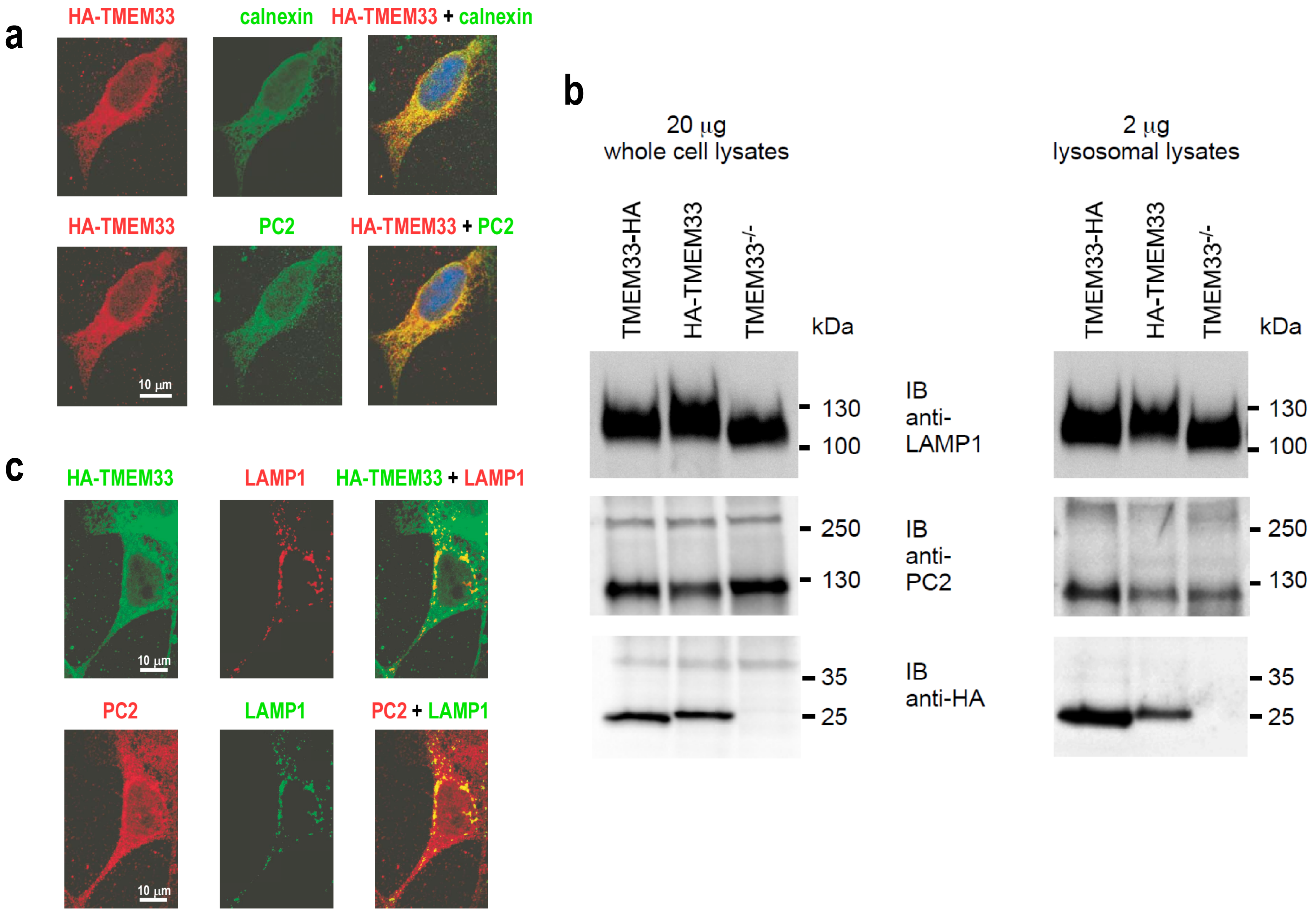

Supplementary Figure 5: Expression of TMEM33 in ER and lysosomes. a) Colocalization of HA-TMEM33 with native PC2 at the ER. TMEM33$^{-/-}$ PCT cells were stably complemented with HA-TMEM33. Top row: HA-TMEM33 is colocalized with calnexin, a marker of the ER. Bottom panel: HA-TMEM33 colocalizes with PC2. In top and bottom right panels co-localization is visible in yellow. **b)** Both TMEM33 and PC2 are detected in purified lysosomes of TMEM33-HA and HA-TMEM33 stably complemented TMEM33$^{-/-}$ PCT cells. LAMP-1 is used as a lysosomal marker. In the left panel 20 μg proteins were loaded for the whole cell lysates (left panel), wheras 2 μg protein was loaded for the lysosomal lysate (right panel). **c)** HA-TMEM33 and native PC2 are co-localized with LAMP1 in lysosomes of TMEM33$^{-/-}$ PCT cells stably complemented with HA-TMEM33. LAMP1 is used as a marker of lysosomes. Source data are provided as a Source Data file.

Supplementary Figure 6

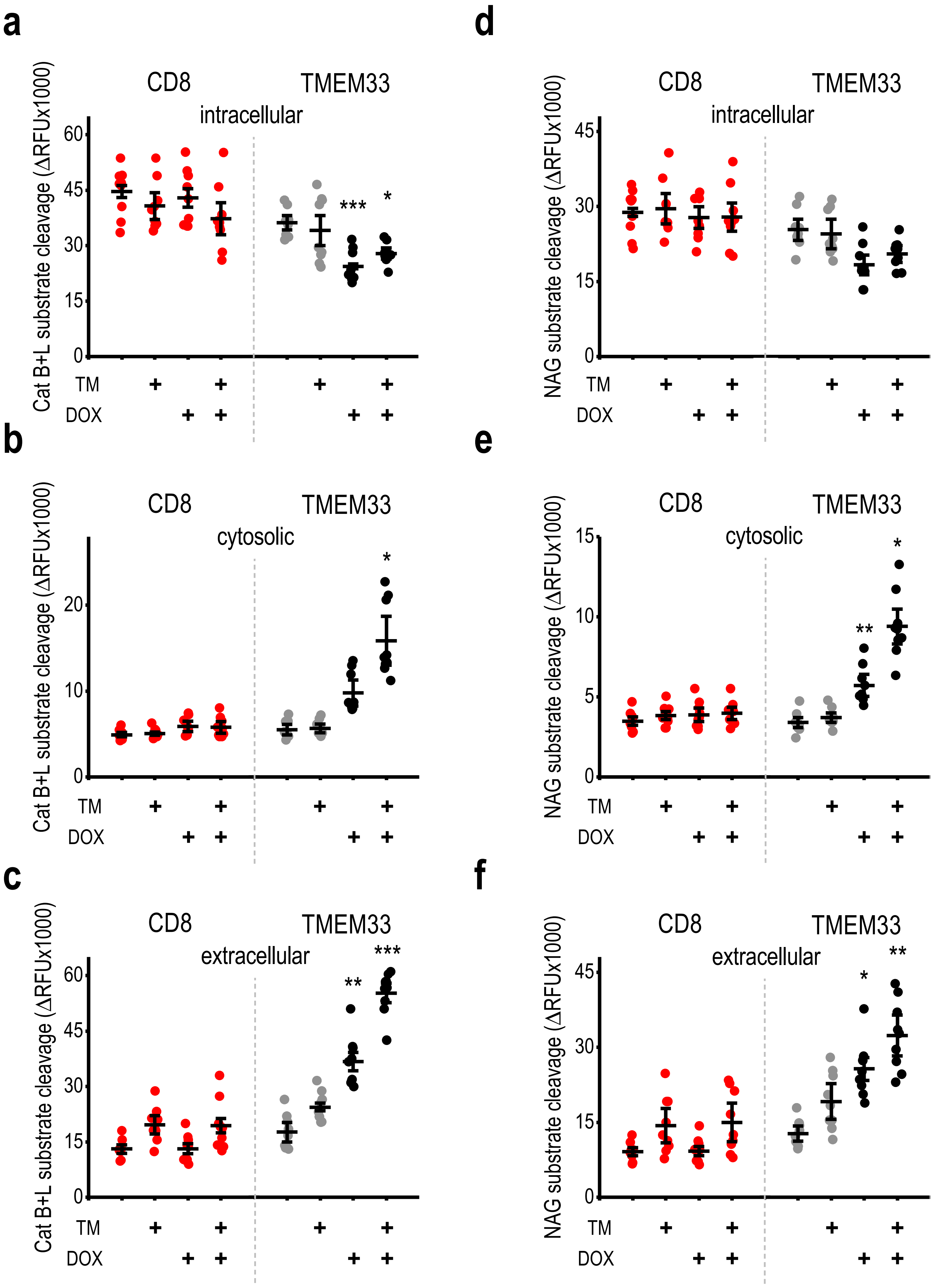

Supplementary Figure 6: TMEM33 induces lysosomal translocation of cathepsins and NAG. Total intracellular cathepsin activity in TMEM33$^{-/-}$ PCT cell lines without or with DOX induced expression of CD8ER (in red) or TMEM33 (in gray or black). RFU: relative fluorescence units. **b)** Cytosolic cathepsin activity. **c)** Extracellular cathepsin activity. **d)** Total intracellular NAG activity. **e)** Cytosolic NAG activity. **f)** Extracellular NAG activity. For panels **e-j**, values represent peak ∆RFUs for $n = 3$ independent experiments done in triplicate. Bars represent mean ∆RFU ± SEM across $n = 3$ experiments overlaid with dot plots. Statistical comparison between non-induced and DMSO treated controls. One star indicates $p < 0.05$, two stars $p < 0.01$ and three stars $p < 0.001$, with two-tailed Student's t test used to evaluate statistical significance. Source data are provided as a Source Data file.

Supplementary Figure 7

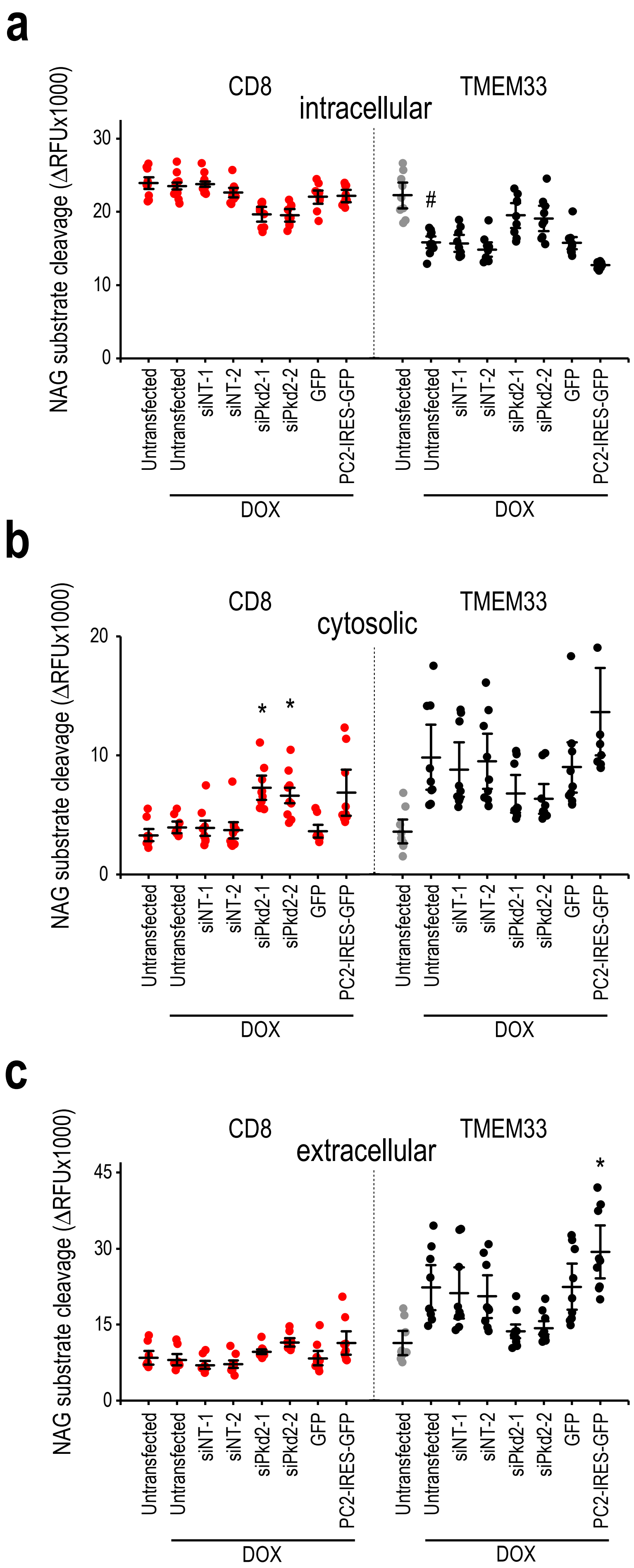

Supplementary Figure 7: TMEM33 induces a *Pkd2*-dependent lysosomal translocation of NAG. a) Effect of *Pkd2* knock down (encoding PC2) by siRNAs or PC2 overexpression on intracellular NAG content activity in TMEM33$^{-/-}$ PCT cell lines without or with DOX induced expression of CD8ER (in red) or TMEM33 (in gray or black). **b)** Same for cytosolic NAG. **c)** Same for extracellular NAG. Values represent peak ∆RFU values from 3 independent experiments done in triplicate. Bars represent mean ∆RFU ± SEM across n = 3 experiments overlaid with dot plots. * compared to respective untransfected DOX induced controls, # compared to respective non-induced controls. One star indicates $p < 0.05$, two stars $p < 0.01$ and three stars $p < 0.001$, with two-tailed Student's t-test to evaluate statistical significance. Source data are provided as a Source Data file.

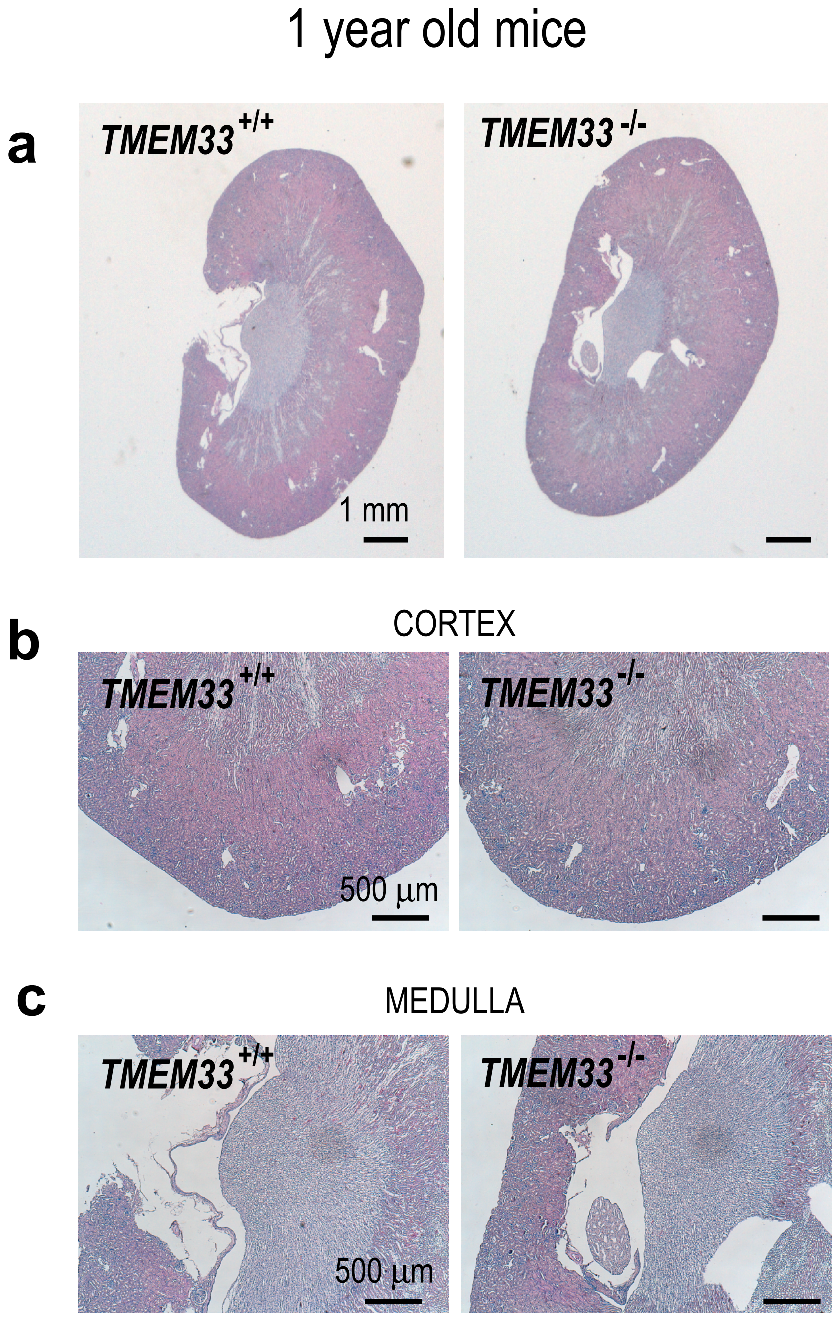

Supplementary Figure 8: Deletion of *TMEM33* does not affect renal morphology in aged mice. a) Renal morphology of 1 year old TMEM33$^{+/+}$ and TMEM33$^{-/-}$ mice. **b**) Cortical area. **C**) Medulla area. Sections were stained with hematoxylin and eosin. Source data are provided as a Source Data file.

Supplementary Figure 9

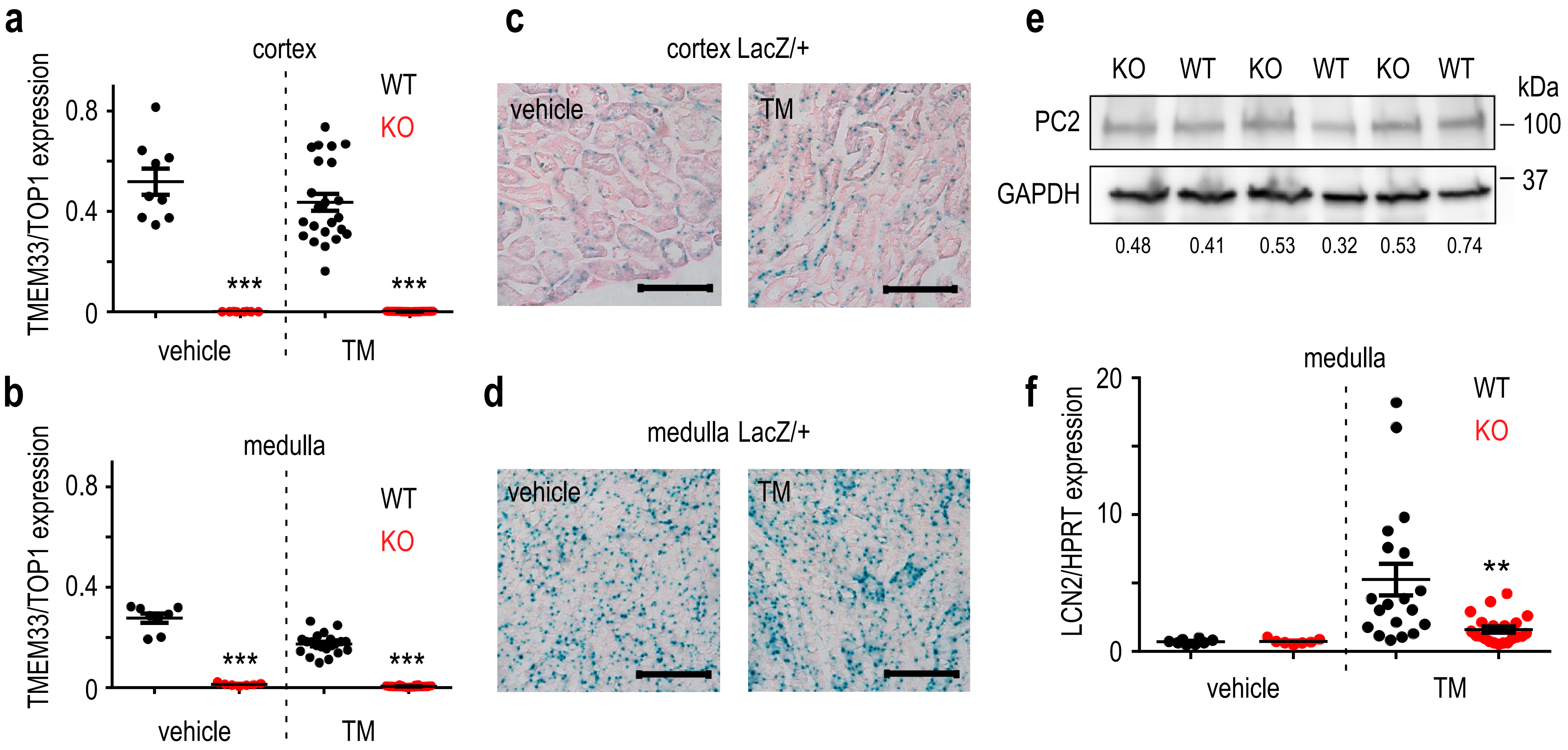

Supplementary Figure 9: TM does not affect TMEM33 expression and its deletion protects renal medulla. a) Expression of TMEM33, as detected by qPCR in the cortex of WT (black dots) or KO red dots) injected with vehicle (DMSO) or TM (2 mg/Kg). **b)** Same for the medulla. **c)** Expression of LacZ (in blue) as a reporter of TMEM33 in the cortical area of $TMEM33^{LacZ/+}$ mice injected with vehicle or TM. Scale bar is 100 μm. **d)** Same for the medulla. **e**) Expression of native PC2 shown by Western blot is not altered in the TMEM33 KO mice. GAPDH expression is shown as a loading control. Normalized PC2/GAPDH values are indicated at the bottom of the blot. **f)** The increase in LCN2 mRNA expression induced by TM injection (2 mg/kg), as detected by qPCR, is blunted in the renal medulla of KO mice. Values are means ± SEM overlaid with dot plots. One star indicates $p < 0.05$, two stars $p < 0.01$ and three stars $p < 0.001$, with a one-way permutation test used to evaluate statistical significance. Source data are provided as a Source Data file.

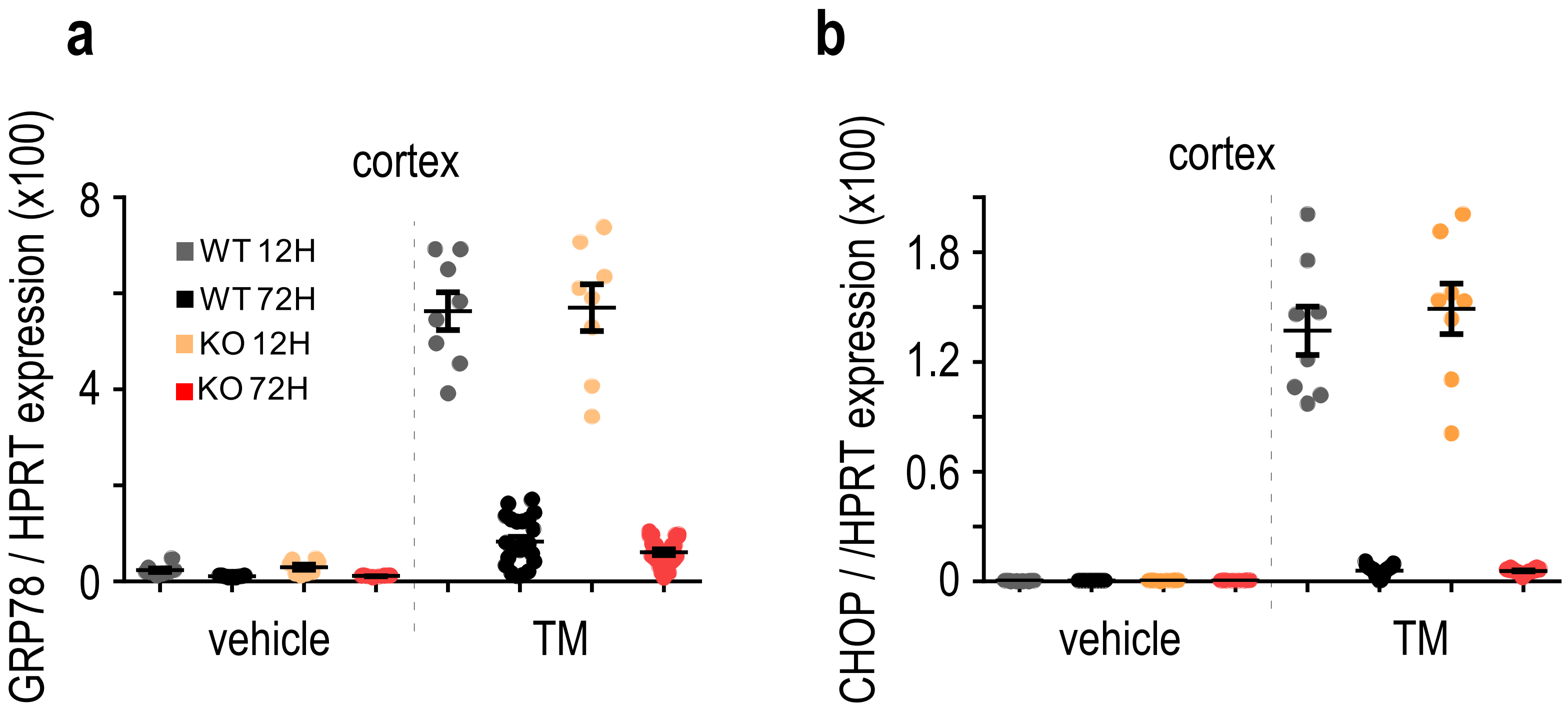

Supplementary Figure 10: ER stress is not affected by *TMEM33* deletion. a) Expression of GRP78, as detected by qPCR in mouse renal cortex 12 (gray and black dots) or 72 hours (orange and red dots) after TM (2 mg/kg) injection, comparing WT and KO. **b**) Same for CHOP expression comparing WT and KO. Values are means ± SEM overlaid with dot plots. A one-way permutation test was used to evaluate statistical significance. Source data are provided as a Source Data file.

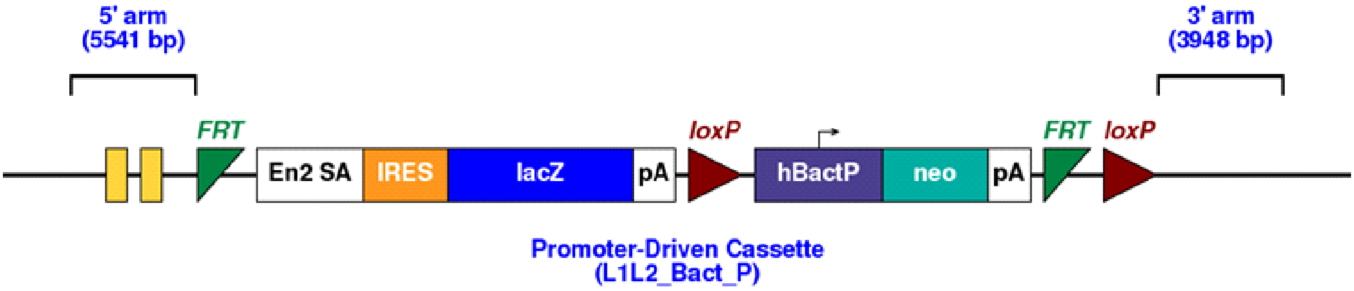

Supplementary Figure 11: DNA construct used to generate the *TMEM33* KO mice. The construct contains a synthetic cassette including LacZ, inserted in intron 2-3. Source data are provided as a Source Data file.

Supplementary Figure 12

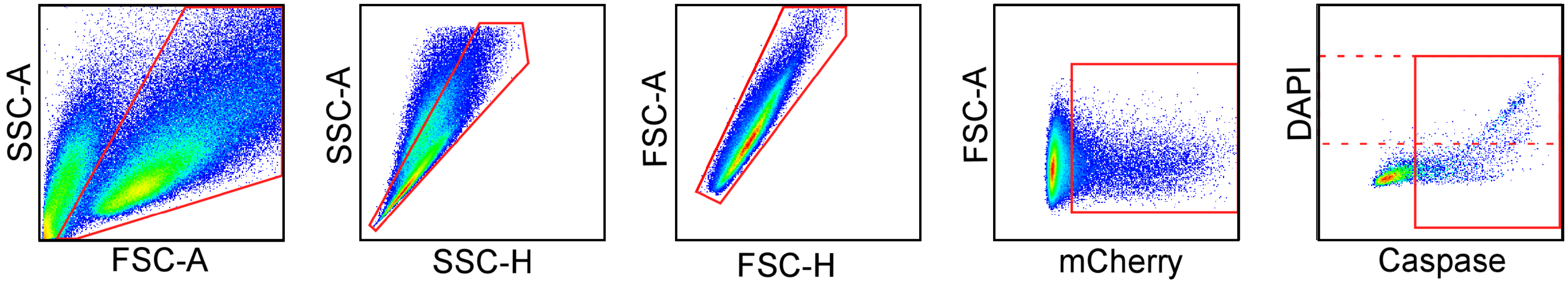

Supplementary Figure 12: FACS gating/sorting. The strategy illustrated for cell sorting is described in the Methods section. Source data are provided as a Source Data file.

Supplementary Table 1: Physiological parameters of WT and (*TMEM33*$^{-/-}$) KO mice blood. Numbers of mice are indicated in the table. Values are means ± SEM. No significant difference was found.

BLOOD	WT (N=4)		KO (N=4)	
	Mean	SEM	Mean	SEM
Body Weight (g)	27.5	0.6	25.7	0.7
pH	7.34	0.02	7.31	0.04
pCO_2 (mmHg)	49.5	2.0	54.4	3.6
pO_2 (mmHg)	29.8	2.2	28.0	3.5
$cHCO_3^-$ (mM)	26.4	1.3	27.6	1.0
Na^+ (mM)	146.5	0.9	145.8	0.5
Ca^{++} (mM)	1.2	0.0	1.2	0.0
Cl^- (mM)	111.3	0.8	110.3	0.3
Hematocrit (%)	42.3	0.7	41.0	0.6
cHgb Hemoglobin (g/dL)	14.4	0.3	13.9	0.2
Glucose (g/L)	1.8	0.2	1.9	0.2
Lactate (mM)	6.4	0.8	7.0	0.7
Creatinine (µmol/l)	32.3	7.1	37.0	4.6

Supplementary Table 2: Physiological parameters of WT and (*TMEM33*$^{-/-}$) KO mice urine. Numbers of mice are indicated in the table. Values are means ± SEM. No significant difference was found.

URINE	WT (N=6)		KO (N=6)	
	Mean	SEM	Mean	SEM
Body Weight (g)	27.2	0.3	25.7	0.3
Na^+ (mM)	234.5	14.0	200.9	10.0
K^+ (mM)	362.1	20.6	315.9	18.5
Ca^{++} (mM)	1.3	0.2	1.0	0.1
Cl^- (mM)	147.3	20.8	146.7	14.7
Urea (mM)	1161.5	141.0	1045.1	69.8
Creatinine (mM)	5.8	0.4	4.6	0.3

Supplementary Table 3: Plasmids used in the yeast two-hybrid system to test for TMEM33/PC2 interaction. These transformations were plated onto glucose – HIS– TRP subsequently streaked onto gal/raf –HIS–TRP–LEU. Yeast colonies that grew on gal/raf –HIS–TRP–LEU plates are indicated by a positive sign in the table.

		Gal/raf-HIS-TRP-LEU
pEG202	pJG4-5	-
pEG202-53	pJG4-5-LTA	+
pEG202-PC2 N term	pJG4-5-TMEM33 N term	-
	pJG4-5-TMEM33 C term	+
pEG202-TMEM33 N term	pJG4-5-PC2 N term	-
	pJG4-5-PC2 C term	+

Supplementary Table 4: Primer sequences for qPCR. Sequences of forward and reverse primers used in this study are listed.

Gene	Primer Forward	Primer Reverse
TMEM33	CCTCTAGAAGAATTCCATATTGTCG	TGGAGACATAGTCTTCTCACAAACA
GRP78	CTGAGGCGTATTTGGGAAAG	TCATGACATTCAGTCCAGCAA
CHOP	GCGACAGAGCCAGAATAACA	TCAGGTGTGGTGGTGTATGAA
LCN2	CCATCTATGAGCTACAAGAGAACAAT	TCTGATCCAGTAGCGACAGC
TRPV4	CCACCCCAGTGACAACAAG	GGAGCTTTGGGGCTCTGT
TOP1	GCCTCCATCACACTACAGCA	TTCGCTGGTACATTCTCATCA
HPRT	CCTCCTCAGACCGCTTTTT	AACCTGGTTCATCATCGCTAA
PKD2	AGGTGTTAGGACGGCTGCT	CCCTGTGGATCTCACTGTCC

Supplementary Table 5: List of antibodies used in the study. Antibodies and dilutions for immune-fluorescence and Western blot experiments are listed.

Experiment	Antibody	Species /Type	Dilution	Cat. No	Manufacturer
Immuno-fluorescence	anti-acetylated tubulin	mouse monoclonal	1 to 200	T7451	Sigma
Immuno-fluorescence	anti-PC2 (E20)	goat polyclonal	1 to 50	sc-10377	SCBT
Immuno-fluorescence	anti-LCN2	goat polyclonal	1 to 75	AF1857	R&D systems
Immuno-fluorescence	anti-LCN2	rabbit polyclonal	1 to 150	AIS12050	Antibody and Immunoassay services, HKU
Immuno-fluorescence	anti-calnexin	rabbit polyclonal	1 to 100	ab22595	Abcam
Immuno-fluorescence	anti-LAMP1	rabbit monoclonal	1 to 50	ab208943	Abcam
Immuno-fluorescence	anti-HA (3F10)	rat monoclonal	1 to 250	ROCHE: 11867423001	Sigma
Immuno-fluorescence	Alexa647 anti-mouse	donkey polyclonal	1 to 1000	A-31571	Invitrogen
Immuno-fluorescence	Alexa594 anti-goat	donkey polyclonal	1 to 1000	A-21447	Invitrogen
Immuno-fluorescence	Alexa488 anti-goat	donkey polyclonal	1 to 1000	A-11055	Invitrogen
Immuno-fluorescence	Alexa594 anti-rabbit	donkey polyclonal	1 to 1000	A-21207	Invitrogen
Immuno-fluorescence	Alexa647 anti-rat	chiken polyclonal	1 to 1000	A-21472	Invitrogen
Western blot	anti-cMYC (9E10)	mouse monoclonal	1 to 1000	ROCHE: 11667149001	Sigma
Western blot	anti-HA (3F10)	rat monoclonal	1 to 1000	ROCHE: 11867423001	Sigma
Western blot	anti-PC2 (E20)	goat polyclonal	1 to 500	sc-10377	SCBT
Western blot	anti-actin (Ac40)	mouse monoclonal	1 to 1000	A4700	Sigma
Western blot	anti-calnexin	rabbit polyclonal	1 to 1000	ab22595	Abcam
Western blot	anti-GAPDH (6C5)	mouse monoclonal	1 to 1000	MAB374	Millipore
Western blot	anti-LAMP1	rabbit monoclonal	1 to 2000	ab208943	Abcam
Western blot	anti-p62	mouse monoclonal	1 to 1000	sc-48402	Santa Cruz Biotechnology
Western blot	anti-LC3B	rabbit polyclonal	1 to 150	NB100-2220	Novus Biologicals
Western blot	anti-Tubulin	mouse monoclonal	1 to 1000	sc-5286	Santa Cruz Biotechnology
Western blot	peroxidase conjugated anti-mouse	donkey polyclonal F(ab')2	1 to 30,000	715-036-150	Jackson ImmunoResearch LABORATORIES, INC.
Western blot	peroxidase conjugated anti-rat	goat polyclonal F(ab')2	1 to 30,000	112-036-062	Jackson ImmunoResearch LABORATORIES, INC.
Western blot	peroxidase conjugated anti-goat	donkey polyclonal F(ab')2	1 to 30,000	705-036-147	Jackson ImmunoResearch LABORATORIES, INC.
Western blot	peroxidase conjugated anti-rabbit	donkey polyclonal IgG	1 to 30,000	711-035-152	Jackson ImmunoResearch LABORATORIES, INC.
