# Supplementary material for: TMEM33 regulates intracellular calcium homeostasis in renal tubular epithelial cells
Source: Nat Commun. 2019 May 2;10:2024. doi: 10.1038/s41467-019-10045-y (PMC6497644; doi:10.1038/s41467-019-10045-y)
Supplement: Supplementary file 3 — Reporting Summary [file 41467_2019_10045_MOESM3_ESM.pdf]

nature research

Corresponding author(s): Eric HONORE

Last updated by author(s): Apr 16, 2019

Reporting Summary

Nature Research wishes to improve the reproducibility of the work that we publish. This form provides structure for consistency and transparency in reporting. For further information on Nature Research policies, see Authors & Referees and the Editorial Policy Checklist.

Statistics

For all statistical analyses, confirm that the following items are present in the figure legend, table legend, main text, or Methods section.

n/a	Confirmed	
☐	☒	The exact sample size (*n*) for each experimental group/condition, given as a discrete number and unit of measurement
☒	☐	A statement on whether measurements were taken from distinct samples or whether the same sample was measured repeatedly
☐	☒	The statistical test(s) used AND whether they are one- or two-sided<br>*Only common tests should be described solely by name; describe more complex techniques in the Methods section.*
☒	☐	A description of all covariates tested
☒	☐	A description of any assumptions or corrections, such as tests of normality and adjustment for multiple comparisons
☐	☒	A full description of the statistical parameters including central tendency (e.g. means) or other basic estimates (e.g. regression coefficient) AND variation (e.g. standard deviation) or associated estimates of uncertainty (e.g. confidence intervals)
☒	☐	For null hypothesis testing, the test statistic (e.g. *F*, *t*, *r*) with confidence intervals, effect sizes, degrees of freedom and *P* value noted<br>*Give P values as exact values whenever suitable.*
☒	☐	For Bayesian analysis, information on the choice of priors and Markov chain Monte Carlo settings
☒	☐	For hierarchical and complex designs, identification of the appropriate level for tests and full reporting of outcomes
☐	☒	Estimates of effect sizes (e.g. Cohen's *d*, Pearson's *r*), indicating how they were calculated

Our web collection on statistics for biologists contains articles on many of the points above.

Software and code

Policy information about availability of computer code

Data collection	Origin 8.0
Data analysis	R Development Core Team: http://www.r-project.org/

For manuscripts utilizing custom algorithms or software that are central to the research but not yet described in published literature, software must be made available to editors/reviewers. We strongly encourage code deposition in a community repository (e.g. GitHub). See the Nature Research guidelines for submitting code & software for further information.

Data

Policy information about availability of data

All manuscripts must include a data availability statement. This statement should provide the following information, where applicable:

- Accession codes, unique identifiers, or web links for publicly available datasets
- A list of figures that have associated raw data
- A description of any restrictions on data availability

Raw data for Fig. 1a-b; Fig. 2; Fig. 3a-b; Fig. 3d; Fig. 4a-g; Fig. 5a-f; Fig. 6; Fig. 7; Fig. 8; Supplementary Fig. 1a-b, d-f; Supplementary Fig. 2e, 2f, 2h, 2j; Supplementary Fig. 3a-d; Supplementary Fig. 4d-f; Supplementary Fig. 5b; Supplementary Fig. 6a-f; Supplementary Fig. 9a, b, e, f and Supplementary Fig. 10 are provided as a Source Data file. All data are available from the corresponding author upon reasonable request.

Field-specific reporting

Please select the one below that is the best fit for your research. If you are not sure, read the appropriate sections before making your selection.

☒ Life sciences ☐ Behavioural & social sciences ☐ Ecological, evolutionary & environmental sciences

For a reference copy of the document with all sections, see nature.com/documents/nr-reporting-summary-flat.pdf

Life sciences study design

All studies must disclose on these points even when the disclosure is negative.

Sample size	When appropriate, significance of the differences was tested with a permutation test (R Development Core Team: http://www.r-project.org/) (n < 30) or with two samples t Test (n > 30).
Data exclusions	no data exclusion
Replication	triplicate transfections were analyzed to ensure reproducibility
Randomization	Allocation was random
Blinding	Investigators were blinded during data analysis

Reporting for specific materials, systems and methods

We require information from authors about some types of materials, experimental systems and methods used in many studies. Here, indicate whether each material, system or method listed is relevant to your study. If you are not sure if a list item applies to your research, read the appropriate section before selecting a response.

Materials & experimental systems

n/a	Involved in the study	
☐	☒	Antibodies
☐	☒	Eukaryotic cell lines
☒	☐	Palaeontology
☐	☒	Animals and other organisms
☒	☐	Human research participants
☒	☐	Clinical data

Methods

n/a	Involved in the study	
☒	☐	ChIP-seq
☐	☒	Flow cytometry
☒	☐	MRI-based neuroimaging

Antibodies

Antibodies used

Mouse monoclonal anti-acetylated tubulin antibody (Sigma T7451; 1/200) and a secondary donkey anti-mouse antibody (Alexa 647 Invitrogen A-31571; 1/500) were used to visualize primary cilia. PC2 (SCBT sc-10377 (E20) antibody 1/50) and a secondary donkey anti-goat (Alexa 594 Invitrogen A-21447, 1/500). LCN2 was detected with antibody from R&D Systems (AF1857) or from Antibody and Immunoassay Services (cat no. 12050), The University of Hong Kong.

For Western blot analysis, mouse anti-c-myc 9E10 (Roche) at 1:1000, rat anti-HA 3F10 (Roche) at 1:1000, rabbit anti-calnexin ab22595 (abcam), mouse anti-GAPDH MAB374 (Millipore) 1:1000, goat anti-PC2 E20 (Santacruz) 1:500 and anti-actin AC40 (Sigma A4700) were used.

Validation

ll antibodies used in the present study have been already published elsewhere.
Citations:
Mouse monoclonal anti-acetylated tubulin antibody (clone 6-11B-1) G Piperno and M T Fuller (1985) The Journal of cell biology, 101(6)
Goat polyclonal anti-LCN2 R&D systems cat no. AF1857 Kim, Y ., et al. (2017) JCI Insight, 2017;2(23):.
Rabbit polyclonal anti-LCN2 AIS cat no. 12050 Yan QW, et al. (2007) Diabetes. 56(10):2533-40.
Mouse monoclonal anti-c-myc (clone 9E10) Roche cat no. 11667149001 Evan G.I., Lewis, G.K., Ramsay,G. and Bishop, J.M. (1985) Mol. Cell. Biol. 5:3610-3616
Rat monoclonal anti-HA high affinity(clone 3F10) Roche cat no. 11867423001 Wilson, I.A., et al. (1984) Cell 37, 767-778
Goat polyclonal anti-PC2 (clone E20) SCBT cat no. sc-10377 Gallagher, A.R., et al. (2000) Proc. Natl. Acad. Sci. USA 97:4017-4022. This antibody was further validated by our group, comparing Pkd2-/- cells transfected with or without HA-tagged PC2
Mouse monoclonal anti-actin (clone AC-40) SIGMA-ALDRICH cat no. A4700 (1988) Lessard, J., Cell Motil. Cytoskel., 10, 349
Rabbit polyclonal anti-calnexin abcam cat no. ab22595 KO VALIDATED Grumati P et al. (2017) Elife 6:.
Mouse monoclonal anti-GAPDH (clone 6C5) cat no. MAB374 Noritake, K., et al. (2015) PloS one 10 e0136952

Eukaryotic cell lines

Policy information about cell lines

Cell line source(s)	Pkd2-/- proximal convoluted tubule cell line was a gift from Dr. Steve Somlo (Yale University, USA). We generated our own TMEM33-/- proximal convoluted tubule cell line conditionally complemented with TMEM33 (see method and results).
Authentication	qPCR
Mycoplasma contamination	negative
Commonly misidentified lines (See ICLAC register)	*Name any commonly misidentified cell lines used in the study and provide a rationale for their use.*

Animals and other organisms

Policy information about studies involving animals; ARRIVE guidelines recommended for reporting animal research

Laboratory animals	C57BL6 breeding at our SPF animal facility
Wild animals	*Provide details on animals observed in or captured in the field; report species, sex and age where possible. Describe how animals were caught and transported and what happened to captive animals after the study (if killed, explain why and describe method; if released, say where and when) OR state that the study did not involve wild animals.*
Field-collected samples	*For laboratory work with field-collected samples, describe all relevant parameters such as housing, maintenance, temperature, photoperiod and end-of-experiment protocol OR state that the study did not involve samples collected from the field.*
Ethics oversight	Experiments were carried out in accordance with the guidelines of the national institutional ethical committee for experimental animals and conform to the European community standards for the care and use of laboratory animals. All manipulations involving animals were carried out under controlled laboratory conditions by qualified personnel. The procedure followed for mouse euthanasia was in strict accordance with the European community standards on the care and use of laboratory animals. Animals were obtained from government-approved animal-raising companies, and a register was kept to record the origin of each animal, all its movements within the laboratory, and the reason for death. This study was approved by our local Committee for ethical and safety issues (CIEPAL-Azur).

Note that full information on the approval of the study protocol must also be provided in the manuscript.

Flow Cytometry

Plots

Confirm that:

☐ The axis labels state the marker and fluorochrome used (e.g. CD4-FITC).

☐ The axis scales are clearly visible. Include numbers along axes only for bottom left plot of group (a 'group' is an analysis of identical markers).

☐ All plots are contour plots with outliers or pseudocolor plots.

☐ A numerical value for number of cells or percentage (with statistics) is provided.

Methodology

Sample preparation	72h after transfection cells are collected, washed and resupend in complete medium supplemented with CellEvent Caspase 3/7 Green detection reagent and DAPI. Samples are incubated at 37°C for 25 minutes and cells were then sorted for mCherry (positive for transfection), DAPI and Caspase activity. For FITC-Annexin V , cells were resuspended in Annexin V buffer . Annexin V and DAPI were added and samples incubated for 15 min at RT. Before sorting, binding buffer was added and cells were then sorted for mCherry (positive for transfection), DAPI and AnnexinV binding.
Instrument	BD LSRII Fortessa, BD Biosciences
Software	BD FACSDIVA™ SOFTWARE
Cell population abundance	Cherry positive cells represent about 10 % of total cell population
Gating strategy	After a first gating using FSC-A/SSC-A, single cells are selected using SSC-A/SSC-H;<br>Then Cherry positive cells were selected. To determine the region we use a negative control corresponding to non-tranfected cells.<br>Among this population gating was done using DAPI and FITC; Regions are defined using control conditions were apoptosis was induced and with or without FITC-Annexin (or CellEvent Caspase 3/7 Green detection reagent)

☒ Tick this box to confirm that a figure exemplifying the gating strategy is provided in the Supplementary Information.
